# The ability of the GENESIS-UV metric to reflect the positive dose-response relationship between cumulative occupational UV exposure and squamous cell carcinoma of the skin

**DOI:** 10.1186/s12995-026-00506-8

**Published:** 2026-03-13

**Authors:** Andreas Seidler, Ulrich Bolm-Audorff, David Reissig, Andrea Bauer, Karla Romero Starke, Sven Connemann, Rolf Ellegast, Peter Knuschke, René Mauer, Henriette Rönsch, Wiho Stöppelmann, Claudine Strehl, Stephan Westerhausen, Marc Wittlich

**Affiliations:** 1 Institute and Policlinic of Occupational and Social Medicine (IPAS), Dresden University of Technology, Faculty of Medicine, Fetscherstr. 74, 01307 Dresden, Germany; 2 Department of Dermatology, University Hospital Carl Gustav Carus, Technical University Dresden, Dresden, Germany; 3 Institute for Occupational Safety and Health of the German Social Accident Insurance, Sankt Augustin, Germany; 4 Institute for Medical Informatics and Biometry (IMB), Faculty of Medicine Carl Gustav Carus, Technische Universität, 01307 Dresden, Germany

**Keywords:** Occupational UV exposure, GENESIS-UV metric, Squamous cell carcinoma of the skin, Case-control study, FB181 study

## Abstract

**Background:**

The newly developed GENESIS-UV metric is based on occupational UV exposure measurements in about 1,000 outdoor-workers (covering 250 different occupational settings). The GENESIS-UV metric calculates occupational exposure values for the standard erythema dose (SED) that differ substantially from those produced by the “Wittlich metric” which applies multiple correction factors to a fixed SED reference value of 300 SED per year. This Wittlich metric has served as the basis for calculating occupational UV exposure of occupational disease No. 5103 (“squamous cell carcinoma or multiple actinic keratoses caused by natural UV radiation”) in Germany since 2015. In a large case-control study (“FB181”, 632 cases and 632 individually matched control subjects), a positive dose-response relationship between UV exposure estimated using the “Wittlich metric” and squamous cell carcinoma (SCC) of the skin could be derived. This study aimed to investigate whether the increased SCC risk for high occupational UV exposure found in the FB181 study can be reflected with the new GENESIS-UV metric.

**Methods:**

Based on propensity-score matched data from the FB181 study, odds ratios (OR) with 95% confidence intervals (CI) for categorized occupational UV exposure were calculated using conditional logistic regression analysis. The analyses were adjusted for age, sex, skin phototype, and non-occupational UV exposure. To examine the suitability of the GENESIS-UV metric, exposure was calculated using both the Wittlich metric and the GENESIS-UV metric. The goodness of fit was assessed using the Akaike information criterion (AIC).

**Results:**

When applying the Wittlich metric, individuals with high cumulative occupational UV exposure (≥ 90th percentile) show a statistically significant increase in OR of 1.95 (95% CI 1.19–3.18). When applying the GENESIS-UV metric, the corresponding OR for reaching or exceeding the 90th percentile is 2.23 (95% CI 1.36–3.65). The goodness of fit of the GENESIS-UV metric is substantially better than that of the Wittlich metric (AIC of 748.0 versus 758.6).

**Conclusions:**

Applying the newly developed GENESIS-UV metric, we were able to confirm the positive dose-response relationship between occupational UV exposure and squamous cell carcinoma of the skin in this sensitivity analysis of the FB181 case-control study. The GENESIS-UV metric proves to be suitable for determining occupational UV exposure.

## Introduction

Non-melanoma skin cancer (NMSC) is the most frequently diagnosed cancer worldwide, accounting for an estimated 7.7 million new cases each year. Approximately three-quarters of these are basal cell carcinomas (BCC) and about one-quarter squamous cell carcinomas (SCC) [1]. In Germany, NMSC likewise represents the most common malignancy, with 222,000 new diagnoses reported in 2019 – predominantly BCC and, to a lesser extent, SCC [2]. Data from the Saarland Cancer Registry show that age-adjusted incidence rates of NMSC have risen more than fourteenfold since 1970, reaching 151.6 cases per 100,000 women and 183.9 per 100,000 men in 2021 [3].

The International Agency for Research on Cancer (IARC) classified solar radiation as carcinogenic to humans in 1992, a conclusion reaffirmed in 2012 [4, 5]. Since 2015, “Squamous cell carcinoma or multiple actinic keratoses of the skin caused by natural UV radiation” has been officially recognized in Germany as occupational disease No. 5103 [6]. In 2023, it ranked as the third most frequently acknowledged occupational disease, with 5,045 recognized and 25 fatal cases [7].

While systematic reviews have provided clear evidence of a SCC hazard from long-term occupational UV exposure [8, 9], to date, there have been very few studies describing the dose-response relationship between occupational UV exposure and SCC. The FB181 study [10, 11], which forms the basis for the sensitivity analysis presented in this paper, revealed a positive dose-response relationship between cumulative occupational UV exposure and SCC. However, the underlying UV exposure assessment was rather coarse and relied on a uniform exposure value for all outdoor workers of 300 SED per year based on measurements in 33 outdoor workers during one year [12], which was adopted to the specific work situation using correction factors [13]. Meanwhile the measurement-based GENESIS-UV metric has been developed, which is based on long-term (seven months each in five consecutive years) UV measurements in about 1,000 study subjects [14]. The aim of this sensitivity analysis of the FB181 study is to investigate whether the increased risk of SCC associated with high cumulative occupational UV exposure can adequately be captured with the new GENESIS-UV metric, and to characterize the dose-response relationship.

## Methods

### Definition of cases and control subjects

This study is part of a broader project addressing both basal cell carcinoma (BCC) and squamous cell carcinoma (SCC); results regarding the use of the GENESIS-UV metric in BCC risk analyses will be reported elsewhere. The study design of the FB181 study has been described in detail previously [10, 11]. Briefly, from 2013 to 2015, consecutive cases with first incident SCC (diagnosed within the past 2 years) were identified by outpatient dermatologists as part of a national network at eight German recruitment sites. All potential cases were offered a full-body dermatological examination and a standardized interview by trained interviewers. The response was 79% among cases.

Sex- and age-stratified population control subjects were randomly selected from population registration offices at the eight recruiting sites. The response in control subjects was 21%.

Altogether, 632 patients with histologically confirmed SCC and 996 population-based control subjects aged 30 years or older were recruited. 1:1 propensity score matching using a nearest-neighbor algorithm was applied to minimize potential confounding by age and sex, yielding 632 cases and 632 individually matched control subjects for the present SCC analysis.

### Ultraviolet (UV) exposure assessment

Cumulative occupational as well as non-occupational UV exposure was assessed based on validated standardized interviews. Interviewers were blinded to participants’ case-control status to minimize information bias. The interviewers were instructed to ensure sufficient accuracy of job titles, which was necessary for translating free-text responses into job codes. In cases of uncertainty, interviewers were instructed to seek clarification.

Exposure assessment in the original FB181 study was performed using the Wittlich metric, a structured and calculation-based approach developed to estimate individual lifetime solar UV exposure [11, 13]. The algorithm is based on a mathematical model that uses a fixed reference value – 300 standard erythema doses (SED) – representing the highest annual UV exposure documented among outdoor workers in Germany [12]. This reference value is subsequently multiplied by several correction factors derived from each participant’s occupational history [13]: time-related factors (accounting for working days per week, proportion of working hours spent outdoors and seasonal work), geographical factors (accounting for latitude and altitude), and personal factors (e.g. accounting for protective measures). To apply the algorithm, each participant’s occupational biography is broken down into discrete episodes reflecting periods of consistent job tasks.

Since 2014, the GENESIS-UV measurement system has provided extensive empirical UV exposure data for a wide range of occupational groups [14, 15]. These measurements were collected to establish a comprehensive job exposure matrix (JEM), intended both to support preventive measures and to eventually replace the calculation-based approach of the Wittlich metric. As the underlying algorithm relies heavily on self-reported information and thus is prone to potential recall bias, a JEM offers the advantage of standardized exposure profiles that better reflect “objective” occupational UV exposure, i.e., independent of self-reports. Moreover, GENESIS-UV data revealed that real-life UV exposure for highly exposed occupations can substantially exceed the former reference value of 300 SED per year [14], highlighting the need for updated and more accurate exposure metrics, especially for epidemiological analyses aimed at analysing dose-response relationships for skin cancer.

In this sensitivity analysis of the FB181 study [11], the GENESIS-UV metric was applied for the first time to a large dataset of real-life working histories. The original FB181 dataset contained a large number of occupational episodes and subsections, sometimes more than ten per participant, depending on career complexity. These episodes were first grouped according to the originally reported job titles. Because the naming of occupations was not standardized during the initial data collection, it was necessary to match each reported occupation to the most suitable GENESIS-UV occupation.

For some predominantly indoor or mixed-exposure jobs, no direct equivalent in the GENESIS data existed; these were therefore assigned to exposure categories based on the estimated proportion of outdoor work derived from previous studies and expert ratings (e.g. indoor occupations like shop assistants or office workers were assigned to spend on average 5% of their working time outdoors). Scaled annual exposure for these categories was calculated by multiplying the maximum measured GENESIS-UV exposure value – 591 SED for dock workers – with the assigned proportion of outdoor work.

For each occupational episode of all study subjects, monthly and annual UV exposure was then recalculated, using either the direct GENESIS-UV measurements (when a matching occupation was available) or the scaled exposure category as described above. In a subsequent quality assurance process, large discrepancies between Wittlich algorithm-based exposure estimates and GENESIS-UV-based recalculations were systematically reviewed. Slight modifications of the job episode-specific UV exposure assessments were particularly made when the original work task description contained role-specific information – such as supervisory positions – that indicated a lower proportion of outdoor work than typical for the occupation. Finally, all episode-specific exposures were summed up to derive updated cumulative occupational UV exposure according to the GENESIS-UV metric.

### Potential confounders and statistics

The characteristics of the cases and control subjects are given in Table 1. The mean (SD) age at SCC diagnosis was 71.2 (± 9.5) years; the mean age of control subjects was 69.7 (± 7.9) years. 70.4% of cases and 68.7% of control subjects were male, 29.6% of cases and 31.3% of control subjects female. Most cases (57.1%) and control subjects (52.1%) had a Fitzpatrick skin phototype 2.

**Table 1:** Characteristics of the study population

	Cases with squamous cell carcinoma (*n* = 632)	Control subjects (*n* = 632)
Sociodemographic characteristics		
Age (years)	71.2 ± 9.5 (37–94)	69.7 ± 7.9 (31–83)
Sex		
Men	445 (70.4)	434 (68.7)
Women	187 (29.6)	198 (31.3)
Education		
No graduation	6 (0.9)	3 (0.5)
9 years	307 (48.6)	209 (33.1)
Middle school (10 years)	143 (22.6)	131 (20.7)
High school (12 years)	114 (18.0)	223 (35.3)
Other	62 (9.8)	66 (10.4)
Clinical and anamnestic parameters Fitzpatrick skin type		
1	70 (11.1)	31 (4.9)
2	361 (57.1)	329 (52.1)
3	190 (30.1)	251 (39.7)
4–6	11 (1.7)	21 (3.3)
Immunosuppressant agents (intake), current and/or previous		
Yes	65 (10.3)	37 (5.9)
No	567 (89.7)	595 (94.1)
Positive family history for skin cancer		
Yes	65 (10.3)	42 (6.6)
No	567 (89.7)	590 (93.4)
Migration background		
Yes	19 (3.0)	31 (4.9)
No	613 (97.0)	601 (95.1)
Smoking		
Smokers	61 (9.7)	80 (12.7)
Ex-smoker 1–10 years	28 (4.4)	31 (4.9)
Ex-smokers > 10 years	253 (40.0)	228 (36.1)
Never smokers	290 (45.9)	293 (46.4)
Genesis UV metric data on UV exposure (SED)		
Occupational UV exposure (total)	3,286.5 ± 4,648.4 (0.0–28,380.8)	2,009.1 ± 2,821.4 (0–16,521.8)
Individuals with occupational UV exposure > 0 SED	389 (61.6)	372 (58.9)
Occupational UV exposure for exposed (> 0 SED) people	5,339.5 ± 4,914.3 (28.5–28,380.8)	3,413.3 ± 2,955.1 (25.7–16,521.8)
Wittlich metric data on UV exposure (SED)		
Occupational UV exposure (total)	2,520.8 ± 4,146.1 (0.0–24,460.8)	1,576.4 ± 2,721.9 (0.0–16,598.4)
Individuals with occupational UV exposure > 0 SED	389 (61.6)	372 (58.9)
Occupational UV exposure for exposed (> 0 SED) people	4,095.5 ± 4,635.7 (21.5–24,460.8)	2,678.1 ± 3,105.2 (3.8–16,598.4)

SED = standard erythema dose

Categorical variables are presented as absolute frequencies (percentages), and continuous variables as mean ± SD (range)

**Fig. 1:**
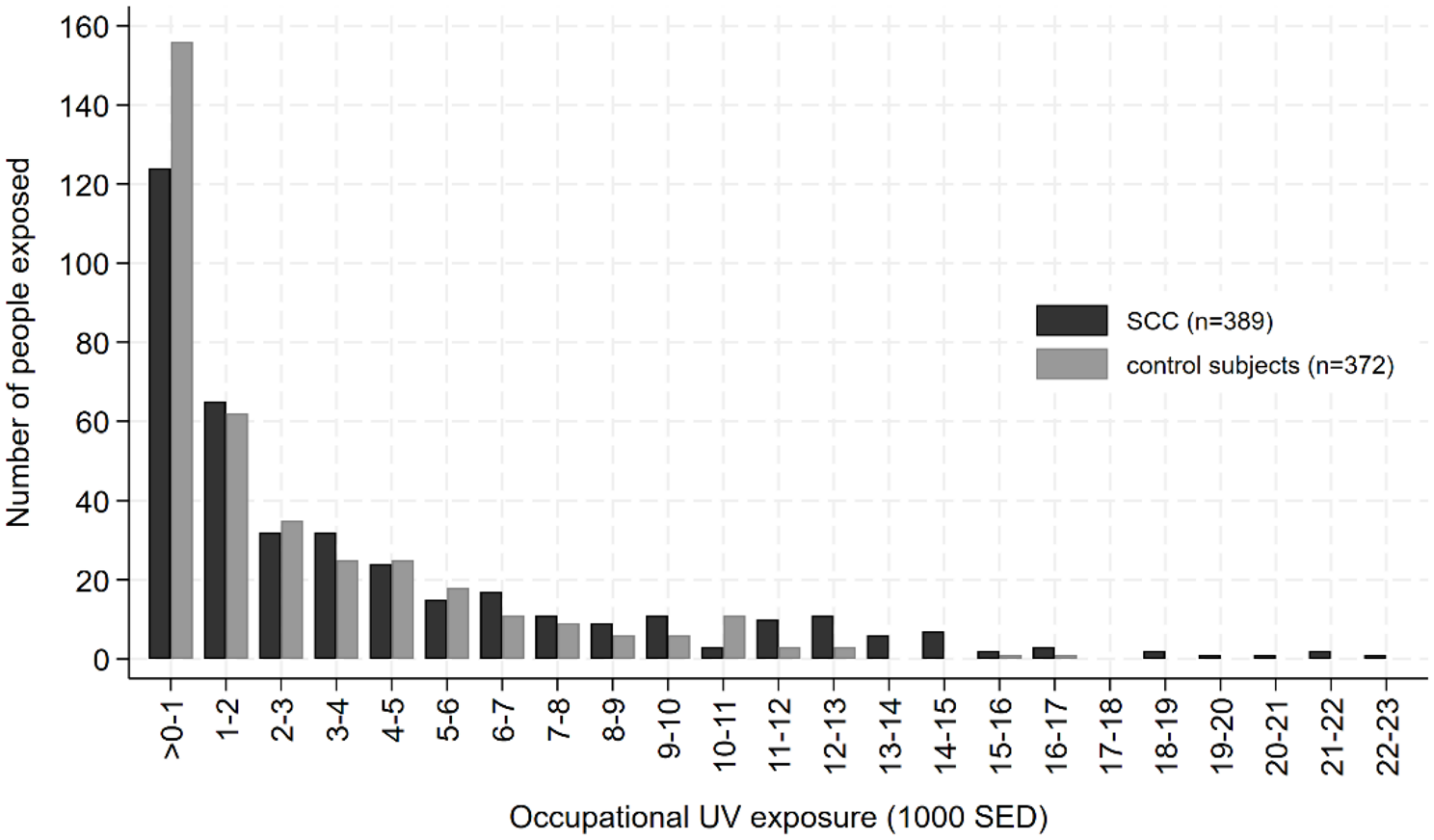
Distribution of cumulative occupational UV exposure according to the Wittlich metric among occupationally exposed (> 0 SED) cases and control subjects

**Fig. 2:**
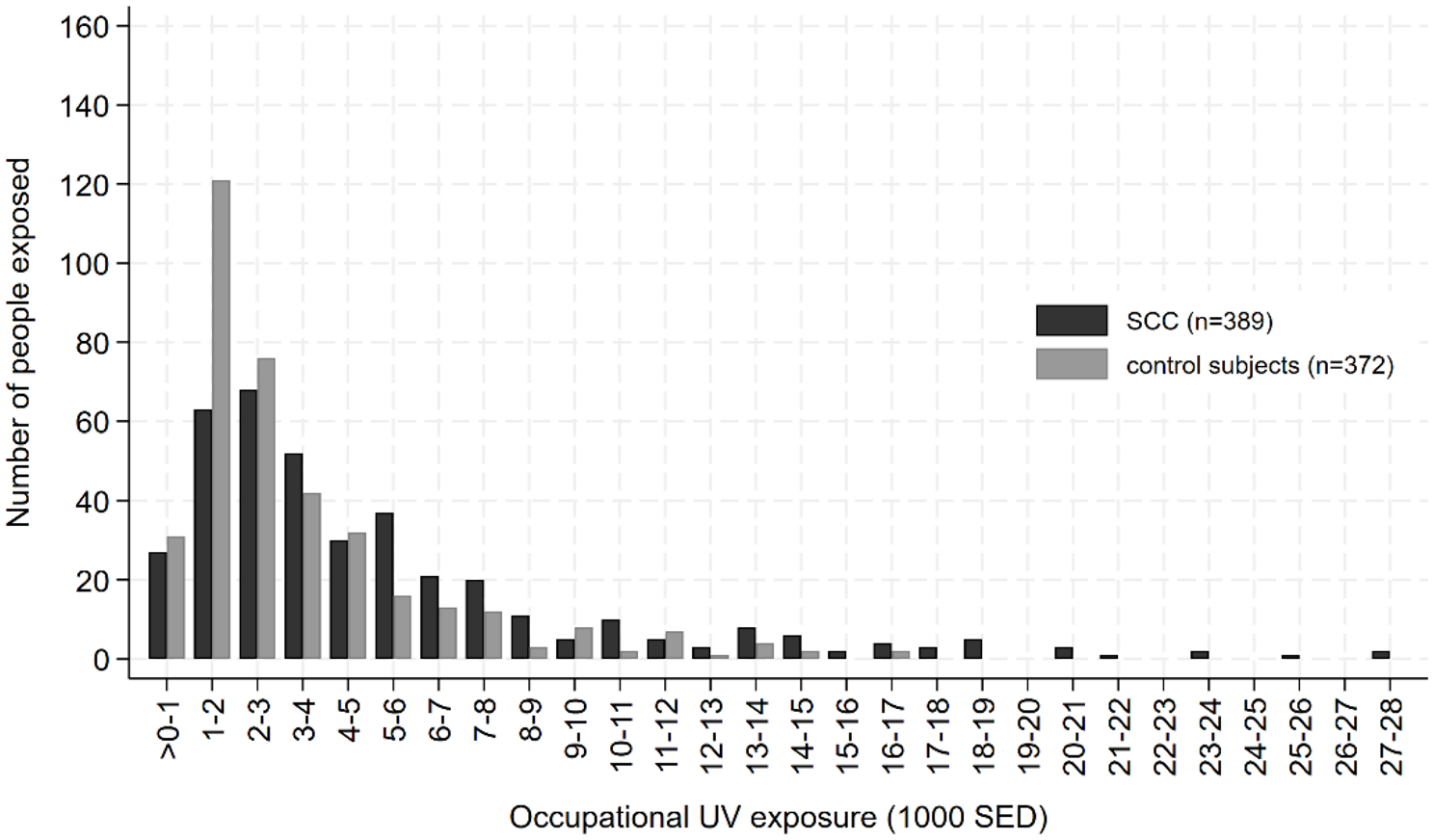
Distribution of cumulative occupational UV exposure according to the GENESIS-UV metric among occupationally exposed (> 0 SED) cases and control subjects

When applying the GENESIS-UV metric, the mean standard erythema dose (SED) was considerably higher in cases (3,286.5 SED) and in control subjects (2,009.1 SED) compared to when applying the Wittlich metric (2,520.8 SED in cases and 1,576.4 SED in control subjects). The distribution of UV exposure among cases with SCC and control subjects is given in Fig. 1 (Wittlich metric) and Fig. 2 (GENESIS-UV metric).

Based on the data from the FB181 study, odds ratios (OR) with 95% confidence intervals (CI) for categorized occupational UV exposure were calculated using conditional logistic regression analysis (40th-59th percentile; 60th-89th percentile and ≥ 90th percentile versus < 40th percentile). To minimize residual confounding, age and sex were included as additional covariates in the logistic regression analysis, in addition to the propensity matching. All analyses were adjusted for age, age squared, sex, Fitzpatrick skin phototype, and non-occupational UV exposure. To examine the suitability of the GENESIS-UV metric, exposure was calculated using both the Wittlich metric and the GENESIS-UV metric. The goodness of fit was assessed using the Akaike information criterion (AIC). A lower AIC value indicates a better model fit.

To examine the dose-response relationship, we included continuous UV exposure as estimated by the GENESIS-UV metric in the conditional logistic regression model. According to Royston & Sauerbrei [16] and Sauerbrei & Royston [17], fractional polynomials use powers from a predefined standard set. We restricted this set a priori to the non-negative subset {0, 0.5, 1, 2, 3} to avoid division by zero and undefined values at zero exposure, and to maintain a parsimonious model structure. For the logarithmic basis function (power 0), we used log(SED + 1) to allow inclusion of subjects with zero cumulative exposure. We selected the best fitting models based on AIC with 95% confidence sets calculated according to Royston & Sauerbrei [16].

At the international level, the doubling of the risk of disease plays an important role for the introduction and recognition of an occupational disease, because the doubling risk is often “translated” into a probability of causation of 50% [18]. We therefore calculated the doubling dose – defined as the exposure that corresponds with the doubling risk – based on the best fitting model.

## Results

**Fig. 3:**
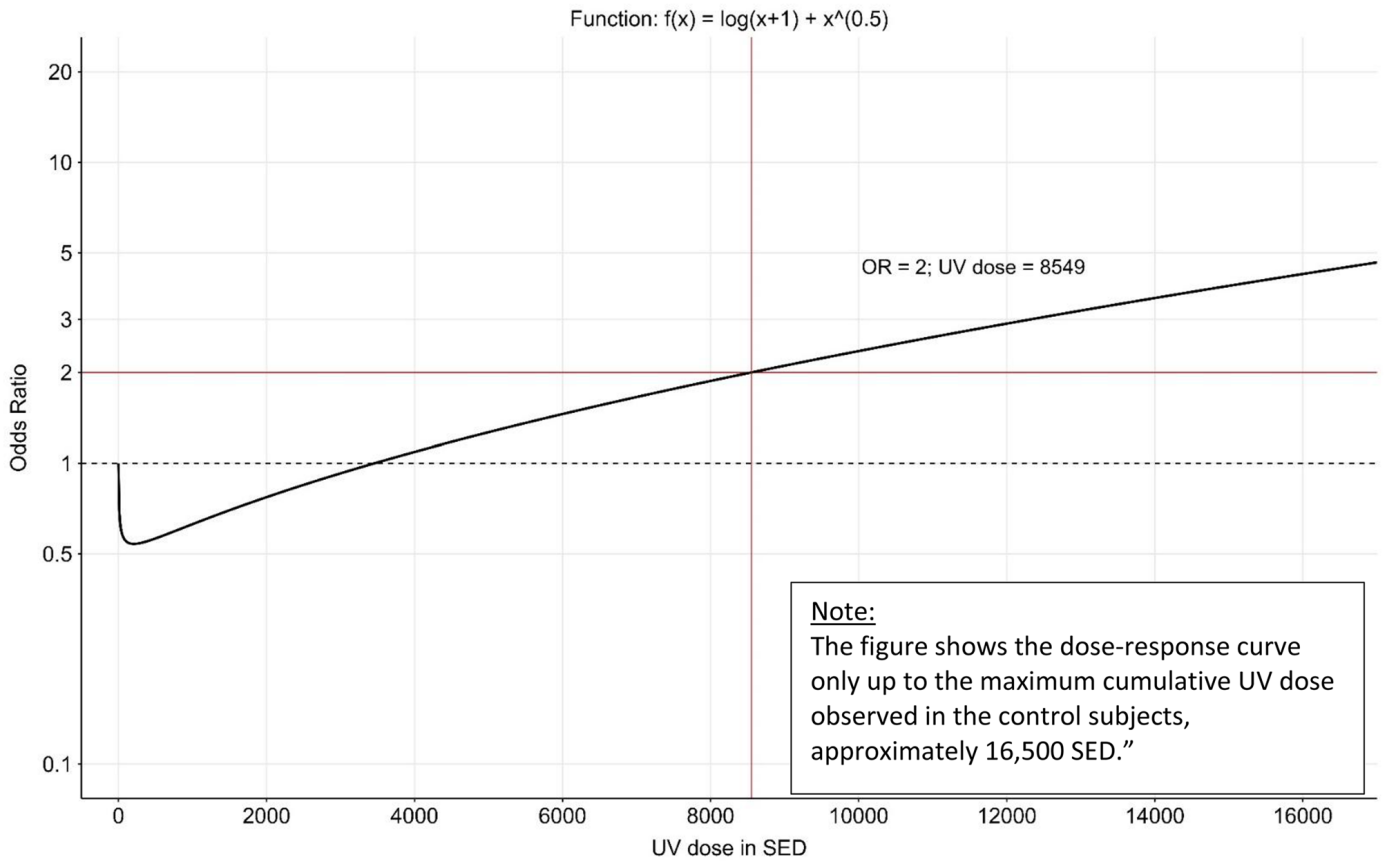
Dose-response relationship between continuous occupational UV exposure (GENESIS-UV metric) and SCC according to the “best model”

**Fig. 4:**
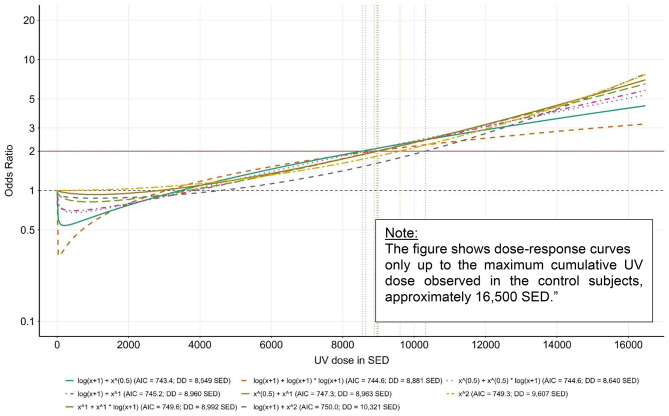
Dose-response relationship between continuous occupational UV exposure (GENESIS-UV metric) and SCC according to the eight statistically indistinguishable “best models” (vertical lines indicate doubling doses)

Odds ratios (OR) for the association of cumulative occupational UV exposure and SCC are given in Table 2. For high cumulative occupational UV exposure (≥ 90th percentile versus < 40th percentile [no UV exposure]), we found a larger risk elevation based on the Genesis-UV metric (OR = 2.23; 95% CI 1.36–3.65) than based on the Wittlich metric (OR = 1.95; 95% CI 1.19–3.18). The goodness of fit was substantially better when applying the GENESIS-UV metric (AIC = 748.0) than when applying the Wittlich metric (AIC = 758.6). When, in a combined analysis, we included both the continuous GENESIS-UV dose and the continuous Wittlich metric dose as first- and second-degree fractional polynomials in the logistic regression analysis, we revealed eight best-fitting models (see Table 3), all of which were based on the GENESIS-UV metric. The lowest AIC value (743.4) could be found when we included log (SED + 1) + Square Root(SED) as a continuous exposure variable in the logistic regression model. Based on this dose model, the doubling dose for SCC was 8,549 SED (Table 3; Fig. 3). The doubling dose for the other seven “best models” within the 95% confidence set was comparable (between 8,640 and 10,321 SED, Fig. 4).

**Table 2:** Cumulative occupational UV exposure and SCC risk estimates (OR) according to Wittlich metric and GENESIS-UV metric

FB181 case-control data (Wittlich metric)				GENESIS-UV metric			
	Cases, *n* (%)	Control subjects, *n* (%)	OR (95% CI)^a^		Cases, *n* (%)	Control subjects, *n* (%)	OR (95% CI)^a^
Occupational UV exposure				Occupational UV exposure			
< 40th percentile (< 12.6 SED)	243 (38.4)	262 (41.5)	Reference	< 40th percentile (0 SED)	243 (38.4)	260 (41.1)	Reference
40–59th percentile (12.6–863.9 SED)	109 (17.2)	144 (22.8)	0.80 (0.56–1.13)	40–59th percentile (> 0–2,076.5 SED)	94 (14.9)	161 (25.5)	0.56 (0.39–0.81)
60–89th percentile (864.0–6,834.7 SED)	195 (30.9)	185 (29.3)	1.01 (0.75–1.36)	60–89th percentile (2,081.0–7,100.8 SED)	206 (32.6)	173 (27.4)	1.19 (0.88–1.63)
≥ 90th percentile (≥ 6,834.8 SED)	85 (13.5)	41 (6.5)	1.95 (1.19–3.18)	≥ 90th percentile (≥ 7,107.1 SED)	89 (14.1)	38 (6.0)	2.23 (1.36–3.65)
**AIC = 758.6**				**AIC = 748.0**			

OR, odds ratio; CI, confidence interval; SED, standard erythema dose. ^a^Adjusted for age, age squared, sex, phototype and non-occupational UV exposure

**Table 3:** GENESIS-UV metric-based dose models and SCC – “Best models” based on Akaike’s Information Criterion (AIC) and 95% confidence set

Function	AIC	AIC (delta)	Doubling dose (SED)
log(x + 1) + x^(0.5)	743.43	0.00	8,549
log(x + 1) + log(x + 1) * log(x + 1)	744.57	1.14	8,881
x^(0.5) + x^(0.5) * log(x + 1)	744.60	1.16	8,640
log(x + 1) + x^(1)	745.15	1.72	8,960
x^(0.5) + x^(1)	747.31	3.87	8,963
x^(2)	749.29	5.86	9,607
x^(1) + x^(1) * log(x + 1)	749.58	6.14	8,992
log(x + 1) + x^(2)	750.03	6.59	10,321

^1^First- and second-degree fractional polynomials of the continuous exposure, using non-negative exponents from the standard exponent set {0, 0.5, 1, 2, 3} based on Royston & Sauerbrei (2008). Dose functions were included in conditional logistic regression analyses, adjusted by age, age squared, sex, phototype, and non-occupational UV exposure

## Discussion

In a sensitivity analysis of the large FB181 case-control study on occupationally acquired skin cancer, we were able to confirm the positive dose-response relationship between occupational UV exposure and squamous cell carcinoma of the skin, applying the newly developed GENESIS-UV metric. The goodness of fit improved when applying this new exposure metric, and the risk estimates for high cumulative occupational UV exposure were comparable to previously published results.

### Strengths and limitations of this study

For the first time, the suitability of the newly developed GENESIS-UV metric to reflect UV-related occupational skin cancer risks could be examined (and approved) in a large population-based case-control study. Our results underline the GENESIS-UV matrix’s purpose to provide a comprehensive (i.e., covering all occupational groups), accurate, and valid assessment of previous and present occupational UV exposure.

The results of the underlying FB181 case-control study may be biased by a low response among control subjects and corresponding socioeconomic differences. To assess whether this potential bias had a substantial effect on skin cancer risk estimates in the FB181 study, in a previously published analysis on the BCC risk estimates (including a control group considerably overlapping with that used in the SCC evaluation), we additionally adjusted the BCC risk estimates for school education [19]. As a result, the BCC risk estimates decreased slightly but remained statistically significantly elevated at high UV exposure. Furthermore, selection bias might have been introduced by slight differences in the catchment areas of cases and control subjects, as the FB181 study also included cases from a somewhat more rural surrounding area. Exclusion of these cases from rural areas had no substantial influence on the BCC risk estimators; therefore, no substantial selection bias can be assumed in this respect [19]. Moreover, the FB181 study included subjects from both western and eastern Germany. In particular, recreational UV exposure in the former GDR may have differed considerably from that in western Germany. However, BCC risk estimators did not differ substantially in a sensitivity analysis stratified by eastern and western Germany [19]. Overall, we demonstrated in this previous publication [19] that the aforementioned potential biases did not substantially affect BCC risk estimates and could not explain the observed positive association between cumulative occupational UV exposure and BCC. These potential biases are therefore unlikely to have substantially affected the SCC risk estimates. Moreover, the present sensitivity analysis compares the results of two different measures of exposure, leaving the analytical approach unchanged. The comparison between the two UV exposure measurement tools should not have been affected by the before-mentioned potential biases. Given the higher SCC risk estimates and the improved goodness-of-fit obtained with the new GENESIS-UV metric, we consider these findings scientifically robust.

However, there are also some limitations concerning the use of the GENESIS-UV metric. Long-term UV measurements were carried out on a relatively small convenience sample of 1–54 employees per occupation (median 8). In 20 out of 76 occupations (26%), UV measurements were only carried out on one to three employees, limiting the generalizability of the obtained UV measurements. However, we would like to emphasize that the development of the GENESIS-UV metric was based on a particularly large number of employees from prevalent occupations. Therefore, in our FB181 case-control study, 86% of occupational episodes with assigned GENESIS-UV measurements could be linked to occupations or suboccupations represented by more than three subjects with UV measurements, and 64% were linked to occupations or suboccupations represented by at least ten subjects.

Nonetheless, it is of particular importance that for the occupations in which occupational UV exposure was determined for 1–3 employees, UV measurements were conducted on a relatively high number of 20–206 working days per occupation [14]. To further elucidate the agreement of GENESIS-UV measurements within occupational subgroups, we calculated the intraclass correlation coefficient (ICC) using a one-way random-intercept model with occupational subgroup specified as a random effect for which at least two (maximum 32) subjects were available. The ICC provides a measure of the agreement between the measured values of different subjects within an occupational subgroup. For the GENESIS-UV metric, an ICC of 0.37 was observed, indicating a relevant, though not dominant, cluster structure. While occupation accounts for a substantial proportion of the exposure variability, at the same time, within-occupation heterogeneity predominates. According to classical reliability criteria, an ICC < 0.40 [20] or < 0.50 [21] would fall within the range of poor reliability. However, these thresholds primarily refer to test-retest and inter-rater reliability. For the present research question, variance decomposition is decisive: an ICC of 0.37 implies that more than one third of the total variability is systematically differentiated between occupational groups and subgroups. In fact, a slightly higher level of agreement can be assumed, as the assignment of UV exposures was not primarily based on the individual subject level, but rather on the intermediate step of calculating monthly values. Subjects for whom UV measurements were available only for individual months may therefore have contributed to an underestimation of the actual agreement within the sub-occupations in this simplified approach.

The limited agreement within occupational subgroups should not primarily be interpreted as a reflection of the relatively small number of individuals with UV measurements per occupational subgroup: when the analysis was restricted to occupational subgroups with more than three individuals, the ICC remained virtually unchanged. Rather, it can be assumed that interindividual heterogeneity in UV exposure also exists within the occupational subgroups. This is attributable, among other factors, to different workplaces and to varying levels of protection of the workplaces against UV radiation. Such heterogeneity would likely not be substantially reduced even with a considerably larger number of individuals with UV measurements, but only through further differentiation of occupational tasks.

Therefore, a larger database would still be desirable as a basis for a job exposure matrix (JEM) on UV radiation. We would like to point out that, even in the recently published EURO-JEM, 64.5% of UV workday measurements are attributable to the GENESIS data [22]. These findings demonstrate that the GENESIS-UV study represents by far the most important dataset on occupational UV exposure in Europe. In the EURO-JEM, measurements were conducted on only one to three subjects in 8 of 49 occupational groups (16.3%) [22]. This indicates that further research in this area is still needed at the international level. However, each JEM can only represent the average exposure for occupations, resolved as finely as possible. In principle, a JEM cannot account for variation in exposure within an occupation. Insofar as the limited assessability of individual occupational exposure may have led to an under- or overestimation of UV exposure, the resulting non-differential bias would tend to slightly underestimate SCC risks. Overall, we consider our finding of a clear positive dose-response relationship to be robust.

Another potential information bias might result from the fact that the UV measurements reflect recent working conditions, whereas participants’ careers span several decades. However, we would like to point out that task- and occupation-specific UV patterns have shown structural stability over decades. To validate this, we consulted sector-specific experts for each occupational field. These experts defined occupation-specific tasks and further assessed how exposure scenarios may have changed over past decades and whether such changes have materially affected overall exposure. The overarching conclusion of the consulted sector-specific experts was that, while individual activities may have shifted, these changes generally have limited impact on overall occupational exposure. However, in the medium to long term, the development of a JEM would be desirable that also attempts to specifically capture higher historical exposures (e.g., working as a tractor operator or caterpillar driver without an enclosed cabin). In the FB181 study, job titles for each occupational episode were entered as free text rather than selected from a predefined list. Consequently, many identical occupations appeared under different titles. Some titles were difficult to classify because the available information on job characteristics was too vague. This might have introduced uncertainties in assigning the corresponding GENESIS-UV assessments. Moreover, as GENESIS-UV measurements focused on high-risk occupations for skin cancer, occupations with low UV exposure are underrepresented in the GENESIS-UV-JEM. This required the above-mentioned definition of exposure categories. The estimated proportion of time spent outdoors is based on other measurement projects and on characteristics of related occupations [14], which may introduce specific uncertainties for some occupational tasks. In addition, the reference value used to characterize occupational exposure is based on mean UV measurements from workers wearing dosimeters. This approach introduces two potential sources of bias: it reflects aggregated rather than individual exposure data, and it represents current work practices, which may have changed substantially over past decades. Finally, due to the lack of information on the use of UV protective measures (such as sunscreen, protective helmets or sun hats, and long-sleeved clothing or long pants), high UV exposure may have been overestimated. Altogether, GENESIS-UV metric-related information bias (most likely non-differential) might have distorted the risk estimates toward 1.

### Dose-response relationship between cumulative occupational UV-metric and SCC

In accordance with the literature [4, 5, 8, 9], we were able to confirm the elevated SCC risk associated with cumulative occupational UV exposure. When we calculated the AIC values for the best-fitting models based on the continuous exposure values of the Wittlich metric and the GENESIS-UV metric combined (using first- and second-degree fractional polynomials), all eight best-fitting models were derived from the GENESIS-UV metric, with none based on the Wittlich metric. This underlines the superiority of the GENESIS-UV metric over the Wittlich metric.

As the goodness-of-fit of the before-mentioned eight GENESIS-UV dose models is statistically almost indistinguishable, future studies are required to clarify the exact course of the dose-response relationship. However, regardless of the statistically equivalent representation of the dose-response relationship across eight different dose models, all models yield comparable doubling doses ranging from about 8,500 to about 10,300 SED. Our study results provide a solid basis for further discussion of exposure assessment for SCC as an occupational disease.

Noteworthy is the finding of a risk reduction at low cumulative UV exposures, followed by an increase in risk at levels above approximately 3,500 SED. One possible explanation could be that low occupational UV radiation promotes thickening of the stratum corneum (so-called solar callus), which provides a certain degree of protection against SCC [23]. However, this explanation remains speculative and needs to be confirmed in further studies. Selection bias in the recruitment of study participants cannot be completely ruled out as an alternative explanation.

## Conclusion

In this sensitivity analysis of the FB181 case-control study, we were able to confirm the suitability of the newly developed GENESIS-UV metric to reflect an elevated squamous cell carcinoma risk by occupational UV-exposure. Applying the GENESIS-UV metric, we found a positive dose-response relationship between occupational UV exposure and squamous cell carcinoma of the skin. A cumulative occupational UV exposure of about 8,500 to 10,000 standard erythema doses as assessed by the GENESIS-UV metric doubles the risk of acquiring a squamous cell carcinoma of the skin.

## Data Availability

Data available on request.

## Abbreviations

95%-CI: 95%-confidence interval
AIC: Akaike information criterion
BCC: Basal cell carcinoma
IARC: International Agency for Research on Cancer
NMSC: Non-melanoma skin cancer
OR: Odds ratio
SCC: Squamous cell carcinoma
SED: Standard erythema dose
UV: Ultraviolet
